# Guillain-Barre Syndrome in a Patient With Asymptomatic Coronavirus Disease 2019 Infection and Major Depressive Disorder

**DOI:** 10.7759/cureus.14161

**Published:** 2021-03-28

**Authors:** Nikita Mokhashi, Gowtham Narla, Christine Marchionni

**Affiliations:** 1 Psychiatry, Lewis Katz School of Medicine at Temple University, Philadelphia, USA; 2 Internal Medicine, St. Luke's University Hospital Network, Bethlehem, USA; 3 Psychiatry, St. Luke's University Hospital Network, Bethlehem, USA

**Keywords:** guillain-barre syndrome (gbs), covid-19, substance use disorder, major depressive disorder

## Abstract

Coronavirus disease 2019 (COVID-19) has had a devastating effect on all aspects of society, including the economy, healthcare, and educational institutions. One underrecognized effect of the pandemic is the decline of mental health in our communities. Studies have shown that pandemic-related stress is associated with increased depression and anxiety. In addition to worsening mental health, COVID-19 infection has been shown to have neurological manifestations. We report the case of a 56-year-old woman with a history of major depressive disorder and alcohol use with no recent history of infection or vaccination who presented with hand and foot paresthesias over the past six weeks, 30 lb weight loss, dysphoric mood, and acutely progressive ambulatory dysfunction over the past two weeks, for which she required assistance to ambulate. Psychiatric evaluation was significant for depressive symptoms. On neurologic examination, she had decreased deep tendon reflexes and ataxic, jerky gait. She was found to be positive for COVID-19. Labs and findings demonstrated albuminocytologic dissociation which suggests presumptive diagnosis of Guillain-Barre syndrome, prompting treatment with intravenous immunoglobulin for five days. She was noted to be deficient in zinc, folate, copper, and borderline B-12, as well as mild hyponatremia, hypokalemia, and hypomagnesemia likely secondary to depression-induced loss of appetite and alcohol use disorder. Guillain-Barre is a severe and debilitating outcome that must be considered when evaluating neuromuscular weakness in the setting of COVID-19, even in asymptomatic patients. Our case highlights the multifactorial intersection between Guillain-Barre syndrome, COVID-19, and concomitant mental health and alcohol use disorder.

## Introduction

Coronavirus disease 2019 (COVID-19) has had a devastating effect on all aspects of society, including the economy, healthcare, and educational institutions. One underrecognized effect of the pandemic is the decline of mental health in our communities. Kujawa et al. reported that pandemic-related stress was associated with increased depression and anxiety [1]. Similarly, in a study among 1,041 adults in Ireland, Hyland et al. demonstrated that 28% of the people screened positive for generalized anxiety disorder (GAD) or depression in the first week of the COVID-19 lockdown [2] compared to a baseline lifetime prevalence of 5.1-11.9% of GAD in the United States [3,4]. Stressors during this time include less time with friends and family, deaths of loved ones succumbing to COVID-19, and housing and employment instability [5]. In addition to worsening mental health, COVID-19 infection has been shown to have neurological manifestations, including axonal motor sensory neuropathy, taste and smell impairment, skeletal muscle injury, myositis, Guillain-Barre syndrome (GBS), Bell’s palsy, stroke, seizures, and altered sensorium [6,7]. Here we explore a case that highlights the multifactorial effects of COVID-19 on mental and neurological health.

## Case presentation

A 56-year-old woman with no recent history of infection or vaccination presented with hand and foot paresthesias over the past six weeks and acutely progressive ambulatory dysfunction over the past two weeks, for which she required assistance to ambulate. Associated symptoms included decreased appetite with a 30 lb weight loss over four months, dysphagia with dry heaving, impaired concentration, fatigue, and dysphoric mood. Her medical and social history was otherwise notable for the diagnosis of major depressive disorder (MDD) and increased alcohol intake of four drinks per day over the last year.

Psychiatric evaluation was significant for depressed mood with no suicidal ideation, low energy and concentration, sleeplessness, decreased pleasure from activities, a paradoxical indifference regarding her inability to walk, and normal Montreal Cognitive Assessment test result. On neurologic examination, the following reflexes were bilaterally decreased: biceps, triceps, wrist flexion and extension, grip strength, hip flexion, quadriceps, hamstrings, and plantar- and dorsi-flexion. Gait was ataxic and jerky. No sensory or cranial nerve deficits were seen. Due to the onset and quality of her symptoms, we did not believe that her acute ambulatory complaints were secondary to depression and pursued a further workup.

Computed tomography of the abdomen and pelvis showed hepatic steatosis with no masses. She was found to be positive for COVID-19 and negative for respiratory syncytial virus, influenza A/B, Lyme disease, and West Nile virus. Her only symptom of COVID-19 was mild diarrhea. Vitals were stable with no hypoxia. Table 1 describes relevant labs and findings, demonstrating albuminocytologic dissociation which suggests presumptive diagnosis of GBS. Increased immunoglobulin G (IgG) synthetic rate, and cerebrospinal fluid (CSF)/serum albumin index was also seen, prompting treatment with intravenous immunoglobulin (IVIG) for five days. Vitamins and electrolytes were repleted due to zinc, folate, copper, and borderline B-12 deficiencies, as well as mild hyponatremia, hypokalemia, and hypomagnesemia. Two weeks after the completion of treatment, her ambulation began improving.

**Table 1:** Laboratory findings of the patient. CMP, comprehensive metabolic panel; BUN, blood urea nitrogen; AST, aspartate aminotransferase; ALT, alanine aminotransferase; CBC, complete blood count; MCV, mean corpuscular volume; MCHC, mean corpuscular hemoglobin concentration; WBC, white blood cell; RBC, red blood cell; Hgb, hemoglobin; INR, international normalized ratio; PTT, prothrombin time; ESR, erythrocyte sedimentation rate; IgG, immunoglobulin G; CSF, cerebrospinal fluid

Lab values		Normal ranges		Lab values		Normal ranges
CMP				Protime with INR, PTT		
Na	133	136-145 mmol/L		Protime	14.7	11.6-14.5 seconds
Cl	96	100-108 mmol/L		INR	1.13	0.84-1.19
K	3.5	3.5-5.3 mmol/L		PTT	25	23-37 seconds
BUN	11	5-25 mg/dL		Vitamins/Minerals		
Glucose, random	109	65-140 mg/dL		Folate	1.5	3.1-17.5 ng/mL
Calcium	8.6	8.3-10.1 mg/dL		B1	36.8	66.5-200 nmol/L
AST	58	5-45 U/L		B-12	260	100-900 pg/mL
ALT	47	12-78 U/L		Homocysteine	52.7	3.7-11.2 umol/L
Alkaline phosphatase	94	46-116 U/L		Zinc	31	56-134 µg/dL
Total protein	6.3	6.4-8.2 g/dL		Methylmalonic acid	68	0-367nmol/L
Albumin	2.8	3.5-5.0 g/dL		Copper	68	72-166 µg/dL
Magnesium	1.5	1.6-2.6 mg/dL		Inflammatory markers		
Iron panel and CBC				ESR	6	0-29 mm/hour
MCV	107	82-98 fL		D-dimer	1.18	<0.50 µg/mL FEU
MCHC	35.3	31.4-37.4 g/dL		Procalcitonin	0.1	<=0.25 ng/mL
Ferritin		8-388 ng/mL		Spinal fluid		
WBC	8.23	4.31-10.16 thousand/uL		Appearance	Colorless	
RBC	3.64	3.81-5.12 million/uL		WBC	1/uL	0-5/uL
Hgb	13.7	11.5-15.4 g/dL		Neutrophils %	20	%
Platelet count	141	149-390 thousands/uL		Lymphocytes %	40	%
Differential				Glucose	55	50-80 mg/dL
Neutrophils %	82	43-75%		Protein	73	15-45 mg/dL
Immature granulocytes %	1	0-2%		RBC	0	0-10 uL
Lymphocytes relative	10	14-44%		IgG	5.3	0-8.6 mg/dL
Monocytes relative	7	4-12%		IgG Index	0.6	0-0.7
Eosinophils	0	0-6%		IgG synthetic rate	3.8	-9.9 to +3.3 mg/day
Basophils relative	0	0-1%		CSF/Serum albumin index	10	0-8
Absolute neutrophils	6.75	1.85-7.62 thousands/uL		Oligoclonal bands	Negative	n/a
Lymphocytes absolute	0.86	0.60-4.47 thousands/uL		Myelin basic protein	2.9	0-3.7 ng/mL
Monocytes absolute	0.55	0.17-1.22 thousands/uL		Angiotensin converting enzyme	<1.5	0-2.1 ng/mL
Eosinophils absolute	0.01	0.00-0.61 thousands/uL				
Basophils absolute	0.02	0.00-0.10 thousands/uL				

## Discussion

Depression has been shown to affect physiologic functions, including loss of appetite [8]. With no diagnosed malignancy or other known causes, it is possible that our patient’s depression led to decreased appetite and 30 lb weight loss. On presentation, she was deficient in magnesium, zinc, copper, folate, and borderline B12. Her inadequate nutrition and excessive alcohol intake may have led to these deficiencies and electrolyte abnormalities. Copper, folate, and B12 deficiencies can result in paresthesias and gait abnormalities [9-11]. We suspect that our patient’s mild, chronic ambulatory dysfunction was likely due to nutritional deficiency secondary to depression-induced anorexia and increased alcohol intake. Another important consideration is that her initial mild symptoms could have been an early manifestation of GBS that eventually progressed to paralysis.

In the two weeks prior to presentation, she developed severe ambulatory dysfunction that escalated to an inability to walk independently. Using the Brighton criteria [12], we determined that her acute symptoms were likely due to GBS secondary to COVID-19 infection based on the following findings: CSF studies showing elevated protein at 73 mg/dL and normal cell count, bilateral progressive flaccid weakness of the limbs, bulbar weakness (difficulty swallowing), decreased deep tendon reflexes, COVID-19 illness pattern, and absence of an alternative diagnosis.

During the pandemic, there have been other similar reports. Finsterer et al. performed a retrospective literature review and documented that 62 patients were found to have GBS secondary to COVID-19 [13]. When comparing patients with and without COVID-19, Fragiel et al. found that the odds of getting GBS was higher in COVID-19 patients (odds ratio = 6.30) [14]. Most of the cases reported thus far have been in patients who had COVID-19 symptoms [13,15,16], while our patient only had mild diarrhea. As our patient did not develop respiratory symptoms, it is possible that the mechanism of GBS is para-infectious rather than post-infectious. Other reports of patients with GBS prior to COVID-19 infection or with asymptomatic COVID-19 support this theory [13,17]. It is plausible that the virus can affect the nervous system prior to the respiratory system. Previous studies have shown that the severe acute respiratory syndrome coronavirus 2 (SARS-CoV-2) virus has neuro-invasive effects [18]. The virus has spiked antigens that enter cells via attaching to ACE2 receptors present in the lungs, nasopharynx, kidneys, heart, brain, intestines, vascular endothelium, and testicles [7]. Receptors in the brain suggest that the virus can directly invade and damage the nervous system. However, while SARS-CoV-2 has been found in the CSF in a few patients with GBS, most reports have not documented this finding [13].

Most studies have found that the mean time from COVID-19 infection to GBS is nine to eleven days [14-16]. Due to our patient’s lack of respiratory symptoms, it is unclear when she contracted COVID-19. Based on the onset time of her severe neurologic symptoms, we suspect that she was infected at least six weeks prior to presentation.

We acknowledge that the lack of electrophysiologic studies is a limitation of our report. Due to patient refusal and no anticipated treatment changes with results, we did not perform an electromyogram or nerve conduction studies.

## Conclusions

While most other reports of GBS during the pandemic depict symptomatic COVID-19 patients in isolation, our case describes an asymptomatic patient as well as the effects of depression and alcohol use disorder on symptoms and diagnosis. GBS is a severe and debilitating outcome that must be considered when evaluating neuromuscular weakness in the setting of COVID-19, even in asymptomatic patients. Timely recognition and treatment of this disease with IVIG is essential. Our case highlights the multifactorial intersection between GBS, COVID-19, and concomitant mental health and alcohol use disorder.

## References

[1] Kujawa A, Green H, Compas BE, Dickey L, Pegg S (2020). Exposure to COVID-19 pandemic stress: associations with depression and anxiety in emerging adults in the United States. Depress Anxiety.

[2] Hyland P, Shevlin M, McBride O (2020). Anxiety and depression in the Republic of Ireland during the COVID-19 pandemic. Acta Psychiatr Scand.

[3] Wittchen HU, Zhao S, Kessler RC, Eaton WW (1994). DSM-III-R generalized anxiety disorder in the National Comorbidity Survey. Arch Gen Psychiatry.

[4] Kessler RC, Gruber M, Hettema JM, Hwang I, Sampson N, Yonkers KA (2008). Co-morbid major depression and generalized anxiety disorders in the National Comorbidity Survey follow-up. Psychol Med.

[5] Rudenstine S, McNeal K, Schulder T, Ettman CK, Hernandez M, Gvozdieva K, Galea S (2021). Depression and anxiety during the COVID-19 pandemic in an urban, low-income public university sample. J Trauma Stress.

[6] Neo WL, Ng JCF, Iyer NG (2021). The great pretender-Bell's palsy secondary to SARS-CoV-2?. Clin Case Rep.

[7] Benny R, Khadilkar SV (2020). COVID 19: neuromuscular manifestations. Ann Indian Acad Neurol.

[8] Baxter LC (2016). Appetite changes in depression. Am J Psychiatry.

[9] Kumar N, Gross JB Jr, Ahlskog JE (2004). Copper deficiency myelopathy produces a clinical picture like subacute combined degeneration. Neurology.

[10] Reynolds EH (2014). The neurology of folic acid deficiency. Handb Clin Neurol.

[11] Stabler SP (2013). Clinical practice. Vitamin B12 deficiency. N Engl J Med.

[12] Sejvar JJ, Kohl KS, Gidudu J (2011). Guillain-Barré syndrome and Fisher syndrome: case definitions and guidelines for collection, analysis, and presentation of immunization safety data. Vaccine.

[13] Finsterer J, Scorza FA, Fiorini AC (2021). SARS-CoV-2-associated Guillain-Barre syndrome in 62 patients. Eur J Neurol.

[14] Fragiel M, Miró Ò, Llorens P (2021). Incidence, clinical, risk factors and outcomes of Guillain-Barré in Covid-19. Ann Neurol.

[15] De Sanctis P, Doneddu PE, Viganò L, Selmi C, Nobile-Orazio E (2020). Guillain-Barré syndrome associated with SARS-CoV-2 infection. A systematic review. Eur J Neurol.

[16] Caress JB, Castoro RJ, Simmons Z, Scelsa SN, Lewis RA, Ahlawat A, Narayanaswami P (2020). COVID-19-associated Guillain-Barré syndrome: the early pandemic experience. Muscle Nerve.

[17] Oguz-Akarsu E, Ozpar R, Mirzayev H, Acet-Ozturk NA, Hakyemez B, Ediger D, Karli N (2020). Guillain-Barré syndrome in a patient with minimal symptoms of COVID-19 infection. Muscle Nerve.

[18] Sahin AR, Erdogan A, Agaoglu PM (2020). 2019 novel coronavirus (COVID-19) outbreak: a review of the current literature. Eurasian J Med Oncol.

